# Laparoscopic removal of a very large uterus weighting 5320 g is feasible and safe: a case report

**DOI:** 10.1186/s12893-017-0248-4

**Published:** 2017-05-04

**Authors:** Antonio Macciò, Paraskevas Kotsonis, Fabrizio Lavra, Giacomo Chiappe, Daniela Sanna, Fausto Zamboni, Clelia Madeddu

**Affiliations:** 1 Department of Gynecologic Oncology, Azienda Ospedaliera Brotzu, via Jenner, 09100 Cagliari, Italy; 2 Department of Anesthesiology, Azienda Ospedaliera Brotzu, Cagliari, Italy; 3 Department of General Surgery, Azienda Ospedaliera Brotzu, Cagliari, Italy; 4 Department of Medical Sciences and Public Health, University of Cagliari, Cagliari, Italy

**Keywords:** Laparoscopic hysterectomy, Large uteri, Minimally invasive surgery, Quality of life, Case report

## Abstract

**Background:**

Total laparoscopic hysterectomy (TLH) has demonstrated to be feasible and safe in the presence of very large uteri. However, it is currently difficult to establish the upper uterine weight limit for successful performance of a laparoscopic hysterectomy.

**Case presentation:**

Here we report the case of a TLH performed for a very large fibromatous uteri weighing 5320 g in a 40-year-old Caucasian woman. The surgery had no complications with an operating time of approximately 220 min. The patient was discharged from the hospital on postoperative day 3 in very good condition. To our knowledge, the present paper is the only to describe a uterus of this size removed by laparoscopic hysterectomy.

**Conclusions:**

Our case demonstrates that uterine size is not a determinant for a final surgical decision to use laparoscopic hysterectomy. Therefore, if not contraindicated by the patient’s comorbidities or peculiar anatomical conditions, we believe that laparoscopic hysterectomy could be performed in the presence of large uteri without hypothetical weight limits.

## Background

It is currently well established that total laparoscopic hysterectomy (TLH) has several advantages compared to traditional total abdominal hysterectomy such as faster recovery, less postoperative pain, lower intraoperative blood loss, and improved cosmetic appearance. Until a few years ago, surgeons were undecided about the safe upper weight limit for the appropriateness of TLH for patients with very large fibromatous uteri [1]. Some authors with skill in minimally invasive surgery demonstrated that TLH is feasible for uteri larger than 1000 g [1–3]. In particular, Alperin et al. published a retrospective analysis including women who underwent laparoscopic hysterectomy with uteri ranging from 500 to 4500 g [3]. Furthermore, Kondo et al. [1] demonstrated the feasibility of laparoscopic hysterectomy in a large retrospective series of patients with enlarged uteri ranging from 1000 g to 4660 g. Recently, in a series of prospective data from 461 TLHs including uteri weighing >800 g and the largest weighing 4000 g, we confirmed that the minimally invasive approach was feasible and safe independent of uterine weight [4]. It is therefore currently difficult to determine the uterine upper weight limit for successful performance of a laparoscopic hysterectomy, with consideration for the right technique and experience in all possible cases. Here, we report the case of a TLH performed for a very large fibromatous uteri weighing 5320 g [4].

## Case Presentation

**Fig. 1:**
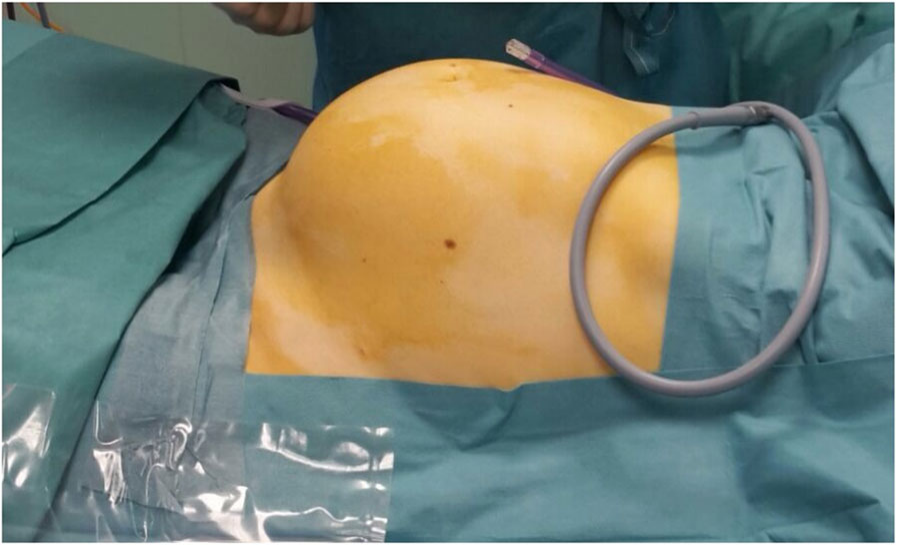
Enlarged abdomen with the patient lying supine before surgery

**Fig. 2:**
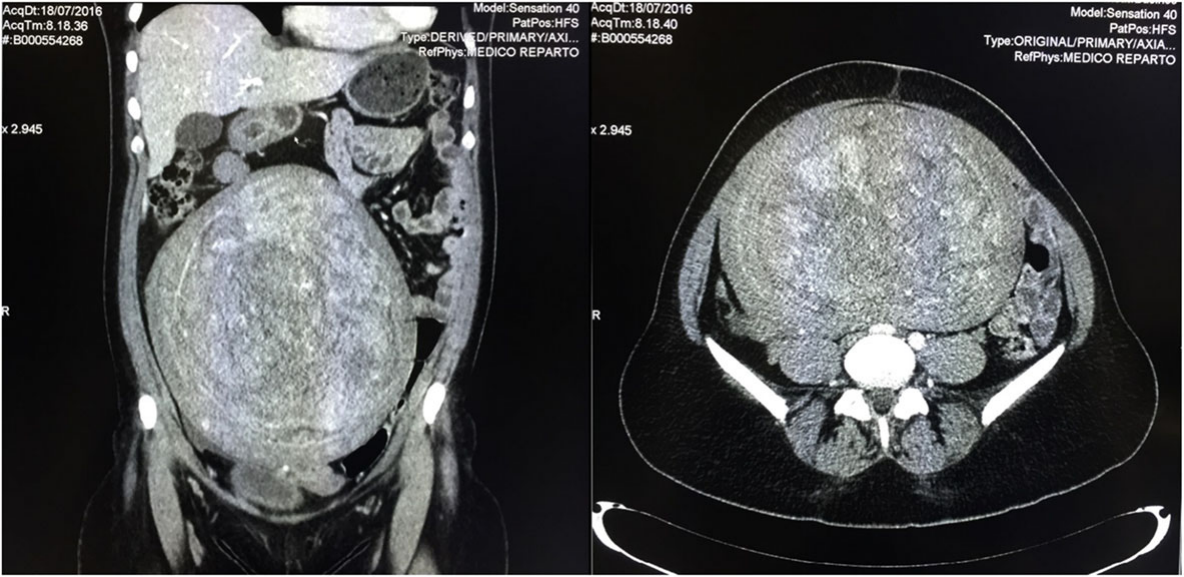
Coronal view and transverse view of computed tomography showing the enlarged uterus occupying the entire abdomen

A 40-year-old, Caucasian nulliparous female presented to our department with the complaint of menorrhagia, worsening dyspnea, constipation, and movement problems. She had a history of a progressive abdominal distension, primarily occurring in the previous 12 months, and a 6-month history of vague abdominal pain and abdominal swelling, which gradually increased over the previous 2 months. She was anemic (hemoglobin, 10.0 g/dl) with normal liver and renal functions. The patient’s past medical and surgical history were unremarkable. Physical and bimanual pelvic examination revealed a pelvic-abdominal mass indicated by palpation to be the uterus extending well cephalad of the third space above the transverse umbilical line and clearly evident by inspection with the patient lying supine (Fig. 1). An ultrasound was performed both abdominally and vaginally, and a large fibroid uterus was confirmed. The patient also underwent a computed tomography (CT) examination that showed a large uterus occupying the entire abdomen (Fig. 2). No hydronephrosis was noted. To rule out possible malignancy prior to the operation, the patient underwent cervico-vaginal smear and endometrial sampling. The Pap smear and endometrial biopsies were negative. The patient was counseled on the various surgical options, about the risks of morcellation of potential occult uterine leiomyosarcoma (LMS) or smooth muscle tumors of uncertain malignant potential, and opted for a minimally invasive approach if possible. Written informed consent was obtained for the procedure as well as for the publication of the case report and the accompanying images.

### Surgical technique

**Fig. 3:**
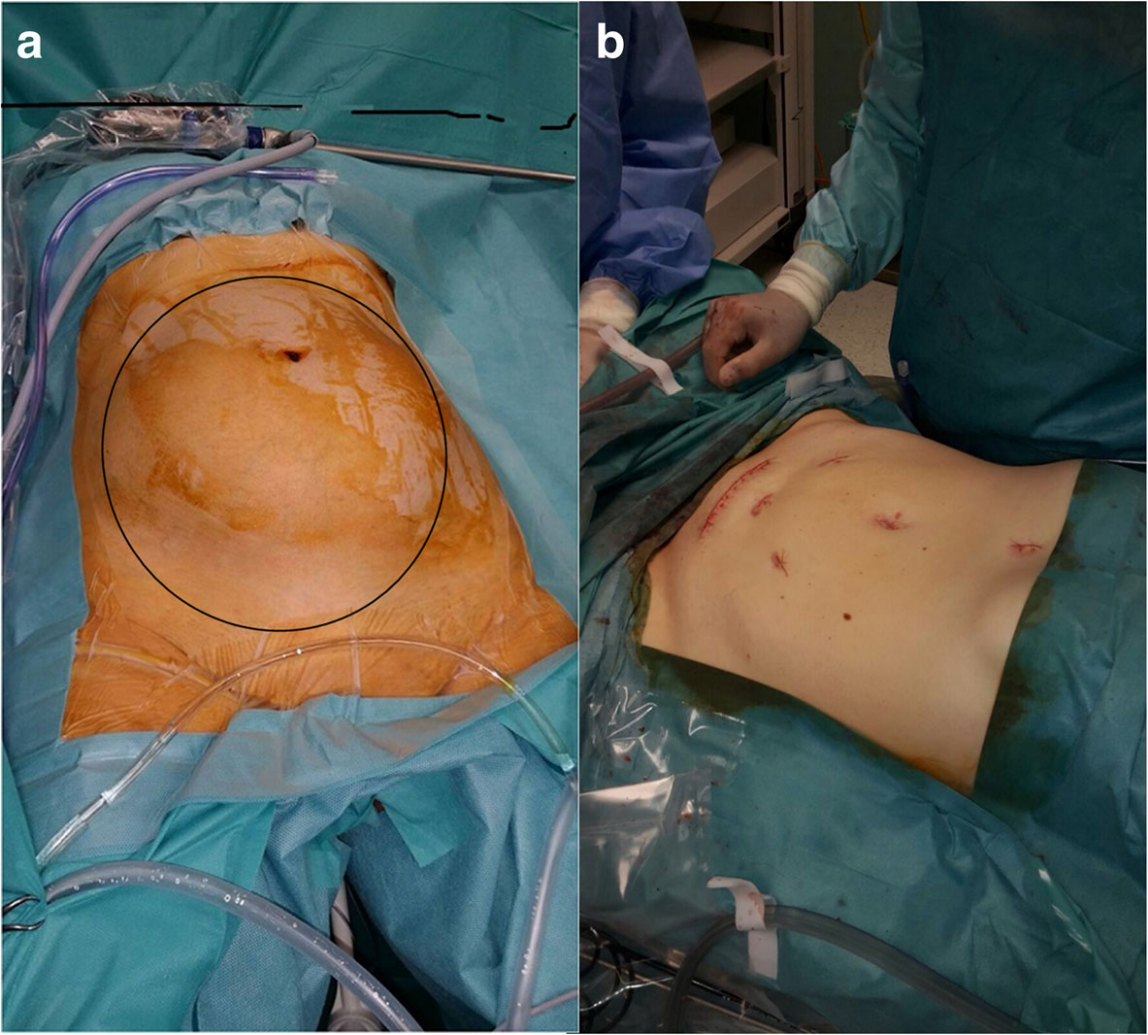
**a** Viewing from above of the abdomen with highlighted (*black circle*) the contour of the large uterus; (**b**) Skin incisions at the end after closure

**Fig. 4:**
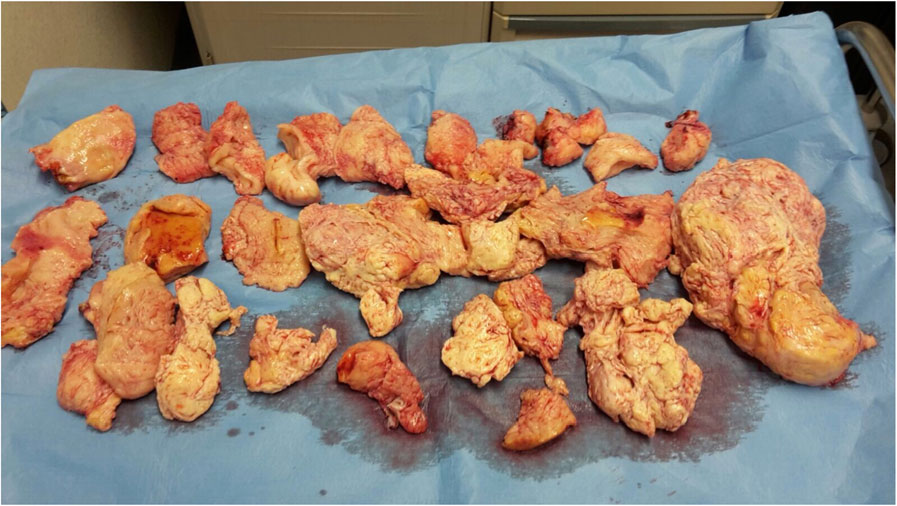
Uterus (final weight, 5320 g) after morcellation

The TLH procedure was carried out according to our previous literature [4]. Thus, bowel preparation was performed by the administration of oral laxatives 3 days prior to surgery and rectal enemas were administered every 8 h before surgery over the previous 24 h. The surgery was performed under general anesthesia with the patient in the lithotomic position. Urinary catheterization was performed and a nasogastric tube was inserted due to the risk of gastric distention and perforation. We used the ClearView Uterine Manipulator-7 cm (Ethicon Endo-Surgery, Cincinnati, USA) to allow for better manipulation of the uterus. A 12-mm trocar was placed by open procedure just below the xiphoid process and a pneumoperitoneum of 10–14 mmHg was created in a neutral position (a supine position where the patient’s spinal column is aligned with legs parallel to the operatory room bed that is inclined of 0 degree) and maintained throughout the surgery. Intra-abdominal visualization was achieved using a 10 mm, 0° telescope (Karl Storz, Tuttlingen, Germany). Two 5-mm trocars were inserted in the hypochondrium lateral to the rectus abdominis muscle, and a third 12-mm trocar at the umbilicus. The surgical table was placed in Trendelenburg position, modulating the angle in accordance with the anesthesiologist’s requirements at various stages of the operation. The first stage of the surgery involved coagulation and transection of the round ligament. We continued the dissection anteriorly from the round ligaments up to the vesico-uterine peritoneal fold to find the correct plane. Then, the utero-ovarian vessels were coagulated and transected. These procedures were performed using Ligasure (Tyco Healthcare; AutoSuture Co., U.S. Surgical Corp., Norwalk, CT). At this time, another 5 mm trocar was inserted in the suprapubic position and the telescope was inserted into the umbilical trocar. Using the uterine manipulator, the uterus was pushed cephalad to recreate the “traction-counter traction” of the lower uterine segment with the help of lateral laparoscopic instruments used for leverage. This elevates the uterine arteries along the lower cervix, and positions them away from the ureters. A bladder flap was incised and the anterior cervical fascia exposed for dissection of the cervix below the cervico-vaginal margin using monopolar forceps. Then, we completed the opening of the posterior leaf of broad ligaments to better expose the uterosacral ligaments. The uterine arteries were skeletonized with the BiClamp LAP forceps (ERBE GmbH, Tubingen, Germany) and monopolar forceps and we then coagulated them with the BiClamp and made the final section with the monopolar forceps. The uterine arteries were pushed downward to expose the cardinal ligament. Then, the cardinal fibers were incised posteriorly to the uterosacral ligaments, and inferiorly, identifying the lowest limit of dissection as the cervico-vaginal margin using Ligasure. The cervico-vaginal margin was laparoscopically visualized and “palpated” with the laparoscopic instruments; the vagina was incised with monopolar scissors at the precise margin of the cervix. Completion of the colpotomy was performed with Ligasure. After the dissection of the cervix, a Foley catheter was placed into the vagina to avoid pneumoperitoneum loss. The vaginal cuff was laparoscopically sutured with the V-Loc wound closure device (Covidien-Medtronic, Minneapolis, MN, USA); we were careful to include the pubocervical vaginal mucosa and uterosacral ligament in the suture to avoid vaginal prolapse. After the extrafascial hysterectomy, the intact uterus was retrieved from the abdominal cavity through a very low transverse laparotomic incision of about 10 cm (Fig. 3) to limit operating time, using a wound protector/retractor (Wound Edge Protector – 3MTM Steri-DrapeTM 1073, Diegem, Belgium) to protect the incision site, and morcellated with a cold blade scalpel externally to prevent spillage. At the end of the surgery, after the closure of the accessory laparotomy, we laparoscopically checked carefully the abdominal cavity and repeatedly washed it thoroughly [5]. There were no complications; the operating time was approximately 220 min. Intraoperative blood loss was about 50 ml subsequent to a small uterus lesion resulting from the introduction of the first lateral trocar. The final weight of the removed uterus was 5320 g (Fig. 4), and the findings of the pathologic examination were consistent with a benign fibroid uterus. The patient was discharged from the hospital on postoperative day 3 in very good condition. The patient follow-up after surgery did not reported any complication connected with the procedure.

## Discussion and Conclusions

To our knowledge, this is the first TLH of a uterus of this size. Normally, we, as well as a growing number of surgeons, attempt to manage all cases of uterine fibroids via a laparoscopic approach, and we decide to convert to an open abdominal approach only if specific abdominal conditions, such as tenacious adhesions syndrome, exist that may influence the result of a minimally invasive technique. This aims to give the patient the best possible care associated with the best achievable quality of life regardless of the size of the uterus, while simultaneously respecting the protection of her body image. As already indicated in our previous work [4], the elements that must be considered in assessing the feasibility of a laparoscopic approach for removal of large uteri are both the characteristics of the patient and the uterus anatomical relationships, without considering hypothetical limits related to the uterine weight. In this regard, in the present paper, we report the case of a patient that underwent laparoscopic hysterectomy for the removal of a 5320 g uterus without side effects and with very good compliance. A previous study by Guan X et al. in 2014 [6] described a case of hand-assisted laparoscopic hysterectomy with the removal of a 5200 g uterus. The surgery lasted 5 h, and the patient was discharged to home the second day after surgery with an unremarkable recovery. It must be highlighted that Guan X et al. [6] used a GelPort to allow the repetitive entry of an assistant’s hand to help in retraction and manipulation of the uterus. In any case, we believe that the procedure used by Guan X, et al. [6] should also be considered as a mini-invasive procedure in consideration of uterus size, as the author himself pointed out very well in his paper. In 2011, Kondo et al. [1] reported on the laparoscopic removal of a 4660 g uterus in a retrospective series that compared the laparoscopic to the laparotomic hysterectomy for large uteri over 1000 g. He showed that in selected patients, with a careful pre-operative evaluation of anatomical limits, the laparoscopic hysterectomy was successfully completed and resulted in a superior postoperative course compared with that of the laparotomic approach [1]. Subsequently, in a series of large uteri with the largest one weighing 4500 g, Alperin et al. [3], showed that the laparoscopic approach was feasible and that increased uterine weight was not associated with increased operative morbidity.

The procedure in our patient lasted approximately 220 min, as reported by the computer records of the operating room, where about 90 min were devoted to the total hysterectomy and the remaining time to perform the manual morcellation with the extraction of the uterus. In considering studies on laparoscopic hysterectomy for large uteri reporting that increased uterine size is associated with an increase of procedure time [4, 7, 8], our case had an acceptable operative time. Noteworthy, in the present case the timing spent for the hysterectomy was limited, whilst the greatest time was dedicated to the extraction of the huge uterus that required also a lot of attention in performing morcellation, avoiding spillage; then, it was the extraction that influenced significantly the time of the entire operation.

Notably, as well explained by Wu KY et al. [9] and Yazucan et al. [10], and also the case in our patient, the trocars positioning on the basis of the uterine size was the first and valued as the most important step affecting the laparoscopic surgery outcome, in addition to the choice of instruments and the experience and the harmony of the operating team [1, 11]. Furthermore, the techniques that we found helpful in completing the procedure through a minimally invasive approach were changing the trocars sites of the laparoscope throughout the procedure for better visualization (in fact, in the present case we started with the trocar positioned near the xiphoid process and then continued with umbilical access), and as specified by Yazucan et al. [10] the ability to suspend from the pelvic floor the uterus through a skillful use of the uterine manipulator thus obtaining also both the best inspection of the ureter projections and a clear visualization of the vesico-uterine fold that aids to avoid potential bladder injury. Additionally, in our experience the importance of the use of the BiClamp for coagulation of the uterine vessels is relevant: such an instrument provides excellent hemostatic control allowing for good dissection based on the surgical plans. In conclusion, TLH for large uteri by experienced laparoscopists is safe and feasible if some technical strategies are strictly followed [10]. Therefore, as demonstrated by the present case, uterine size is no longer a determinant as a final surgical decision to use laparoscopic hysterectomy. Then, if not contraindicated by the patient’s comorbidities or peculiar anatomical conditions, we believe that laparoscopic hysterectomy could be performed in the presence of large uteri without hypothetical weight limits.

## Abbreviations

CT: Computed tomography
TLH: Total laparoscopic hysterectomy
